# A simple method for *in situ*-labelling with ^15^N and ^13^C of grassland plant species by foliar brushing

**DOI:** 10.1111/j.2041-210X.2010.00072.x

**Published:** 2011-06

**Authors:** Birgit Putz, Thomas Drapela, Wolfgang Wanek, Olaf Schmidt, Thomas Frank, Johann G Zaller

**Affiliations:** 1 Institute of Zoology, University of Natural Resources and Life Sciences ViennaGregor Mendel Straße 33, A-1180 Vienna, Austria; 2 Department of Chemical Ecology and Ecosystem Research, University of ViennaAlthanstraße 14, A-1090 Vienna, Austria; 3 UCD School of Agriculture, Food Science and Veterinary Medicine, University College DublinDublin 4, Ireland

**Keywords:** carbon, foliar labelling technique, IRMS, native grassland species, nitrogen, stable isotope tracers, urea

## Abstract

**1.**Labelling plants with ^15^N and ^13^C stable isotopes usually require cultivation of plants in isotopically enriched soil and gas-tight labelling chambers - both approaches are not suitable if one aims to investigate *in situ* species interactions in real plant communities.

**2.**In this greenhouse experiment, we tested a labelling method in which dual-labelled (^15^N, ^13^C) urea solution is brushed directly onto leaves of twelve temperate grassland species representing grasses, non-leguminous forbs and legumes.

**3.**Across all plant species, shoots (^15^N: 0·145; ^13^C: 0·090 atom percent excess, APE) and roots (^15^N: 0·051; ^13^C: 0·023 APE) were significantly enriched after five daily labelling events. Generally, isotopic enrichments were significantly higher in shoots than in roots. No clear pattern of absolute isotopic enrichment was observed between plant functional groups; however, grasses showed a more even allocation between shoots and roots than forbs and legumes. Isotopic enrichment levels after 4 weeks were lower, higher or unchanged compared to those of week one and varied between species or plant parts.

**4.**Considering the consistent enrichment levels and simplicity of this method, we conclude that it can be applied widely in ecological studies of above-belowground plant–plant or plant–animal interactions even in real plant communities.

## Introduction

Stable isotope labelling is a powerful, quantitative technique used in current ecological research to validate and complement studies at natural abundance levels, for example to elucidate nutrient cycles and organismic interactions within ecosystems (Michener & Kaufman 2007). When studying food webs that involve plants, a common approach is to introduce isotopically enriched plant material into the system and trace elements derived from them in the other food web components (e.g. Simard *et al.* 1997; Herman *et al.* 2000; Martens *et al.* 2001; Hood-Nowotny & Knols 2007; Seeber *et al.* 2009). However, to produce isotopically labelled plant material, plants are usually cultivated in sophisticated labelling chambers for continued release of ^13^CO_2_ that are often not available in ecological laboratories (Berg *et al.* 1991). Pulse-labelling, in which plants are exposed periodically to labelled CO_2_, circumvents many of the logistical constraints and even allows labelling outside of the laboratory, although airtight labelling chambers are still needed (Bromand *et al.* 2001; Leake *et al.* 2006; Subke *et al.* 2009).

Recently, Hertenberger & Wanek (2004) compared ^15^N labelling efficiencies of several alternative methods (e.g. root feeding, stem infiltration, leaf tip feeding, vacuum infiltration, surface abrasion) on three plant species (forbs: *Brassica napus* L., *Centaurea jacea* L.; grass: *Lolium perenne* L.). Generally, their results showed marked differences in plant labelling effectiveness, both with respect to the method applied and the plant species used. Leaf vacuum infiltration and leaf surface abrasion resulted in the lowest ^15^N enrichments of roots and shoots (<1% APE, atom percent excess), while root feeding and stem infiltration (8% APE in shoots and 15% in roots) achieved the best results. Overall, stem infiltration effectively ^15^N labelled plants with thicker stems, while feeding via cut leaf tip was most effective for graminoid plants.

Another frequently used alternative, namely spraying isotopically labelled urea onto the plant surface, has been applied to various crop plants (Schmidt & Scrimgeour 2001; Rasmussen *et al.* 2007; Wichern *et al.* 2007; El-Naggar *et al.* 2008). However, while spraying generally seems to work well, it has the disadvantage that the soil surface needs to be protected to avoid contamination with stable isotopes, thus preventing its use in stands with a dense plant cover where only specific plants need to be labelled. Moreover, it is not known how effective foliar labelling is for various wild plant species and to what extent the isotopic tracer is allocated within the plant.

The objectives of the current study were to test whether (1) dual isotopic labelling with ^15^N and ^13^C applied directly onto the leaf surface is a suitable method for labelling a variety of grassland plant species, (2) isotopic enrichments differ between plant functional groups and between shoots and roots and (3) the persistence of the isotopic label differs between plant species. We conducted a pot experiment in the greenhouse in which 12 plant species comprising three different functional groups (grasses, forbs, legumes) were grown in field soil. We hypothesised that labelling efficacy differs among species according to inherent morphological traits and differences in biomass allocation.

## Material and methods

### Plant and soil material

The experiment was conducted in an unheated greenhouse at the University of Natural Resources and Life Sciences Vienna, from March to June 2008. Foliar isotopic labelling was tested on 12 different plant species that commonly occur in Central European low-fertile grasslands. Test plants included the graminoids –*Arrhenatherum elatius* L., *Briza media* L.*, Bromus erectus* Huds.*, Dactylis glomerata* L.; the non-legume forbs –*Leucanthemum ircutianum* DC.*, Plantago lanceolata* L.*, Rumex obtusifolius* L.*, Salvia pratensis* L.*, Knautia arvensis* Coult.; and the herbaceous legumes –*Lotus corniculatus* L.*, Medicago lupulina* L.*, Trifolium pratense* L. Seeds were obtained from a commercial supplier (Rieger Hofmann GmbH, Blaufelden-Raboldshausen, Germany). We grew one specimen of each plant species individually per pot (3 L volume, 14·5 × 14·5 cm side length, 22 cm height); to ensure regular germination, we initially placed three seeds on the soil surface but later reduced the number of seedlings to one plant per pot. Pots were filled with a 2 : 1 mixture of field soil and quartz sand (quartz sand particle size 1·4–2·2 mm); the mixture had a pH 7·6, *N* = 0·092 g kg^−1^, *P* = 64·5 mg kg^−1^, *K* = 113·6 mg kg^−1^. The field soil was obtained from an arable field of the Experimental Farm of the University of Natural Resources and Life Sciences Vienna, Groß-Enzersdorf, sieved through a 1-cm sieve and sterilized at 120°C for 12 hours before being filled into the pots.

Plants were watered with deionised water when needed; no fertiliser was applied during the experiment. The pots were randomly arranged on a greenhouse table and randomised once a week. We set up 24 replicate pots of each plant species (288 pots in total) and harvested three pots of labelled and three pots of non-labelled controls of each plant species once a week over a period of 4 weeks after the initial labelling event (see below).

### Foliar labelling, harvest and isotopic analysis

For foliar labelling, we prepared a 97 atom%^13^C, 2 atom%^15^N urea solution by dissolving 100 mg 99 atom%^13^C urea and 2 mg 98 atom%^15^N urea (Sigma Aldrich, Vienna, Austria) in 50 mL distilled water. To ensure good contact of the labelling solution with the leaf surface, 12·5 μL wetting agent (Neo-Wett, Kwizda, Vienna, Austria) was added. The control solution consisted of 50 mL distilled water, 102 mg unlabelled urea and 12·5 μL wetting agent. The urea concentrations are similar to those used by Schmidt & Scrimgeour (2001). Attempts to use the labelling solution without the wetting agent were not successful as solution rolled off the leaf surfaces. Labelling started after the plants developed two true leaves: The urea solution was applied with a small paint-brush on the upper and lower leaf surfaces (cotyledons were not labelled); during brushing, leaves were held with forceps. Only small amounts of the solution were applied at a time to avoid contamination of the soil. Brushing the solution was straightforward and usually took only a few seconds per plant individual. Leaves treated with adequate solution had a shiny surface, making it easy to see which leaves were already treated. Labelling was applied once a day over five consecutive days.

Three replicate pots per plant species and treatment were harvested 6 days after the beginning of labelling by carefully excavating the plants and separating roots and shoots. In the following 3 weeks, the remaining replicates were labelled once a week and three replicates per plant species and treatment were harvested 2 days after the last labelling. Over the 4 weeks of our experiment, about 80 mL of labelling solution was used (total leaf area of all labelled plants at the end of the experiment was about 1340 cm^2^). Roots were immediately washed free of soil and dried at 65°C for at least 24 hours. At all four harvesting dates, roots of all legume species showed rhizobia nodules. Shoots were carefully washed, scanned on a flatbed scanner (300 dpi) and afterwards dried at 65°C for at least 24 hours. Leaf area was measured using image analysing software (ImageJ for Windows, Institute of Health, Washington D.C., USA). The dried plant material was ground to a fine powder directly in 2-mL disposable reaction vials using a ball mill (Mixer Mill MM 200, Retsch, Haan, Germany) to avoid cross-contamination of samples and afterwards immediately weighed into tin capsules for isotopic analyses.

Samples were analysed by Continuous-flow isotope ratio mass spectrometry, using an elemental analyzer (EA 1110; CE Instruments, Milano, Italy) coupled to a gas isotope ratio mass spectrometer (Delta Plus; Finnigan MAT, Bremen, Germany). As universal standard for ^15^N we used atmospheric air (*R* = 0·003676), and for ^13^C we used Vienna Pee Dee Belemnite as standard (*R* = 0·0112372). Enrichment of plants post-labelling was calculated by subtracting, for each plant species separately, the mean atom% value of the control plants from the atom% of the labelled plants, yielding atom% excess values (APE).

### Statistical analyses

Because data were not distributed normally even after testing several data transformations, we analysed them using non-parametric tests: Kruskal–Wallis-tests were used to test for significance of differences between means of two or more groups, Mann–Whitney-U-tests were used for pairwise comparisons. Spearman correlations (with Bonferroni correction to control familywise error rate) were calculated for testing the relationships between total plant dry mass and leaf area, and ^15^N enrichment and ^13^C enrichment. All statistical analyses were performed using SPSS 15.0 software (SPSS Inc. Headquarters, Chicago, IL, USA).

## Results

### Plant biomass production and C and N concentrations

With the exception of the grass *B. media*, the test plants grew well during the experiment increasing in biomass on average by 255% from week one to week four (Table S1). Species varied greatly in biomass and C and N concentrations without a clear difference between functional groups (Table S1). Early in the experiment, non-leguminous forbs had the highest biomass followed by grasses and legumes.

### Isotopic enrichment after 1-week foliar labelling

**Fig. 1:**
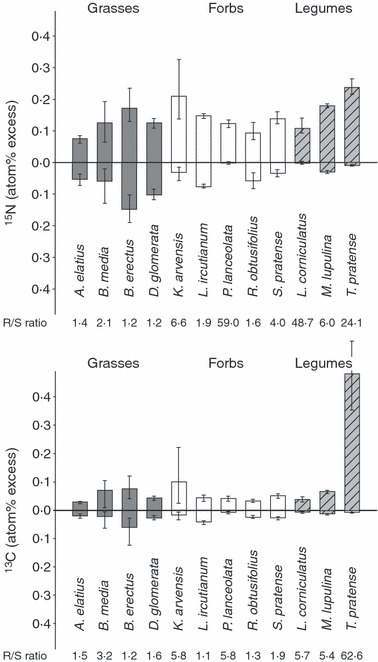
Enrichment in ^15^N and ^13^C (APE) in shoots and roots of 12 grassland species belonging to three functional groups (grasses, non-leguminous forbs, leguminous forbs) after daily foliar labelling for 5 days. Means ± Maximum/Minimum values, *n* = 3. The ratio between enrichments in roots and shoots (R/S ratio) is also shown for each species. [Correction added after online publication 26 Nov 2010: incorrect minus signs removed from *Y*-axes]

Overall, ^15^N isotopic enrichments after 5 labelling events were significantly higher in shoots (0·145 APE) than in roots (0·051 APE; χ^2^ = 26·308, d.f. = 1, *P*<0·001; Fig. 1). Across species, ^15^N enrichment differed marginally significantly in shoots (χ^2^ = 18·199, d.f. = 11, *P*=0·077) and significantly in roots (χ^2^ = 24·237, d.f. = 11, *P*=0·012; Fig. 1). Plant functional groups did not differ in their shoot ^15^N enrichment (χ^2^ = 1·636, d.f. = 2, *P*=0·441); however, grass roots showed a significantly higher mean ^15^N enrichment (0·091 APE) than non-leguminous forbs (0·038 APE; U = 33·000, d.f. = 14, *P*=0·008) and legumes (0·009 APE; U = 5·000, d.f. = 6, *P*=0·002). Legume root ^15^N enrichment was significantly lower than ^15^N enrichment in roots of non-leguminous forbs (U = 15·000, d.f. = 6, *P*=0·026) or grasses (U = 5·000, d.f. = 6, *P*=0·002). Generally, grasses showed a more even allocation of ^15^N enrichment between shoots and roots (shoots average: 0·125 APE; roots average: 0·091 APE) than the other functional groups where more ^15^N was allocated towards shoots (Fig. 1). Average ^15^N enrichment in shoots of non-leguminous forbs was 0·142 APE vs. 0·038 APE in roots. Legumes showed the least balanced allocation of ^15^N enrichment between shoots (0·163 APE) and roots (0·009 APE).

Across species, the enrichment in ^13^C was generally higher in shoots (0·090 APE) than in roots (0·023 APE; χ^2^ = 24·681, d.f. = 1, *P*<0·001; Fig. 1). Comparing all species, the ^13^C enrichment in shoots did not differ among species; however, it differed marginally among species in roots (χ^2^=18·176, d.f. = 11, *P*=0·078; Fig. 1). Functional groups did not differ in their ^13^C shoot enrichment but in their ^13^C root enrichment (χ^2^ = 6·623, d.f. = 2, *P*=0·036). With an average of 0·033 APE, grasses had similar ^13^C root enrichment than non-leguminous forbs (mean = 0·022 APE, U = 78·000, *n* = 12, *P*=0·758); however, ^13^C root enrichment in grasses was significantly higher than that of legumes (mean = 0·008 APE, U = 11·000, *n* = 12, *P*=0·019). Legumes had significantly lower ^13^C root enrichment than non-leguminous forbs (U = 14·000, *n* = 6; *P*=0·021) and grasses (U = 11·000, *n* = 6; *P*=0·019). Similar to ^15^N, the allocation of ^13^C in shoots and roots was more balanced in grasses than in non-leguminous forbs and legumes (Fig. 1).

### Time courses of isotopic enrichments

**Fig. 2:**
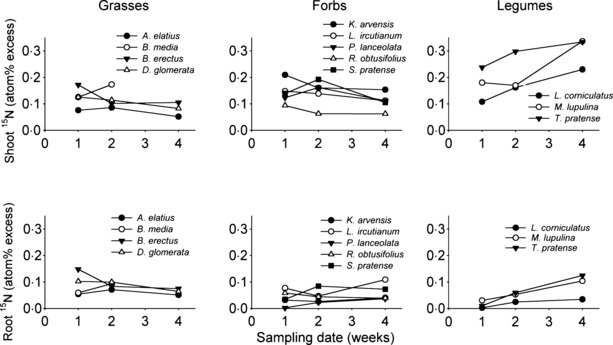
Time course of the ^15^N enrichment in shoots and roots of 12 grassland species comprising the functional groups grasses, non-leguminous forbs and leguminous forbs during 4 weeks of foliar labelling. Means, *n* = 3.

**Fig. 3:**
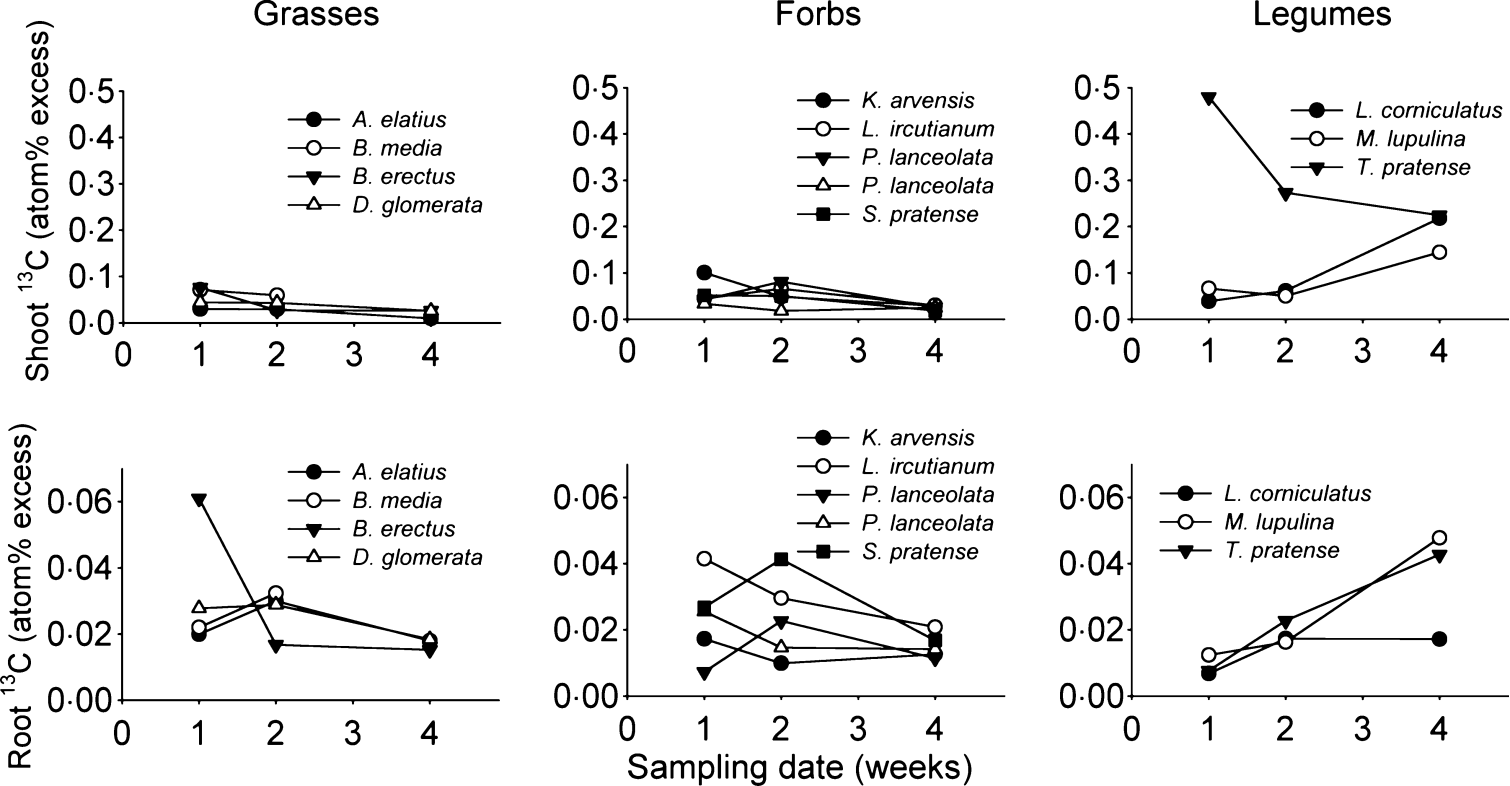
Time course of the ^13^C enrichment in shoots and roots of 12 grassland species comprising the functional groups grasses, non-leguminous forbs and leguminous forbs during 4 weeks of foliar labelling. Means, *n* = 3. Note different *Y*-axis scales for shoots and roots.

Overall, there was considerable ^15^N (Fig. 2) and ^13^C (Fig. 3) enrichment both in shoots and roots even 4 weeks after the first labelling. Averaged across species, grass shoots showed a significant decrease in ^15^N enrichment over the 4 weeks (χ^2^ = 6·102, d.f. = 2, *P*=0·047), while the ^15^N enrichment in grass roots remained unchanged over the 4 weeks (χ^2^ = 3·032, d.f. = 2, *P*=0·220; Fig. 2). Grass ^13^C enrichment significantly decreased over the 4 weeks in shoots (χ^2^ = 12·541, d.f. = 2, *P*=0·002) and roots (χ^2^ = 6·061, d.f. = 2, *P*=0·048; Fig. 3). Non-leguminous forb ^15^N enrichment in shoots and roots remained unchanged over the 4 weeks (χ^2^ = 3·443, d.f. = 2, *P*=0·179 for shoots and χ^2^ = 1·698, d.f. = 2, *P*=0·428 for roots). Non-leguminous forb ^13^C enrichment in shoots decreased significantly (χ^2^ = 10·822, d.f. = 2, *P*=0·004) but remained unchanged in forb roots (χ^2^ = 3·488, d.f. = 2, *P*=0·175). Legume ^15^N enrichment significantly increased in shoots (χ^2^ = 6·045, d.f. = 2, *P*=0·049) and roots (χ^2^ = 12·123, d.f. = 2, *P*=0·002) during the 4 weeks. Legume ^13^C remained unchanged in shoots (χ^2^ = 2·224, d.f. = 2, *P*=0·329) but significantly increased in roots over period of the experimental period (χ^2^ = 13·228, d.f. = 2, *P*=0·001).

### Correlations between isotopic enrichments and plant characteristics

Across all species and dates, ^15^N enrichment of shoots was significantly negatively correlated with shoot dry mass, while ^15^N enrichment of roots was significantly positively correlated with root dry mass (Table 1). Across all species and dates, only shoot ^13^C enrichment was significantly negatively correlated with either shoot dry mass or leaf area, while root ^13^C was unrelated to root dry mass. Among functional groups, both shoot and root ^15^N enrichments were significantly positively correlated with shoot ^13^C or root ^13^C, respectively (Table 1). Only non-leguminous forbs showed statistically significant correlations between ^15^N or ^13^C enrichment and either dry mass or leaf area, while isotope enrichments in grasses and legumes were unrelated to the measured characteristics (Table 1).

**Table 1:** Spearman correlations between ^15^N and ^13^C atom percent excess (APE) isotopic enrichments and plant characteristics across species and sampling dates. Significant correlations (*P* < 0·005) after Bonferroni adjustments are in bold

	Across species	Grasses	Forbs	Legumes
				
Variable	*r*_s_	*r*_s_	*r*_s_	*r*_s_
Shoot ^15^N				
vs. shoot^13^C	**0·823**	**0·880**	**0·629**	**0·792**
vs. shoot dry mass	**−0·498**	−0·122	**−0·588**	−0·073
vs. leaf area	**−0·597**	−0·231	**−0·597**	−0·080
Shoot ^13^C				
vs. shoot dry mass	**−0·466**	−0·236	−0·403	0·197
vs. leaf area	**−0·487**	−0·301	−0·420	−0·197
Root ^15^N				
vs. root^13^C	**0·672**	**0·622**	**0·560**	**0·921**
vs. root dry mass	**0·294**	−0·080	−0·131	0·186
Root ^13^C				
vs. root dry mass	−0·012	−0·221	−0·225	−0·110

## Discussion

To the best of our knowledge, this is the first study demonstrating that brushing ^15^N and ^13^C urea onto the leaf surface of a dozen of grass, non-leguminous forb and legume species is a feasible method for *in situ* dual-labelling of herbaceous plant species. Moreover, the positive correlation between ^15^N and ^13^C enrichment for both shoots and roots indicates that the tested species can successfully be labelled with the two isotopes. In contrast to other studies in which stable isotope solutions were sprayed onto the leaf surface (Below *et al.* 1985; Palta *et al.* 1991; Schmidt & Scrimgeour 2001; Yasmin, Cadisch, & Baggs 2006), our brushing method has the advantage of being more controlled, precise and targeted. Therefore, selected plant species, even in dense stands, can be labelled without unintentionally contaminating other plants or soil. Further, most of the previous studies on foliar labelling were conducted on crop species (maize: Below *et al.* 1985; Schmidt & Scrimgeour 2001; wheat: Palta *et al.* 1991; chickpea: Yasmin, Cadisch, & Baggs 2006), the present study has established that grassland species with much lower growth rates can also be successfully labelled via foliar feeding.

Our expectation that functional groups would significantly differ in their isotopic enrichments because of their morphological differences was only partly met. While isotopic enrichments in shoots were similar between functional groups, the allocation of ^15^N tracers into roots was highest in grasses followed by forbs and legumes, while ^13^C enrichment was similar in grasses and forbs, although higher than in legumes. This indicates that functional groups mainly differed in their allocation of the labelled urea acquired through leaves within the plant. A higher ^15^N and ^13^C allocation into the root systems of the four grass species compared with the non-leguminous forbs and legumes may be explained by the different root system of grasses (Fitter 1987) comprising more homogenous fine roots and a lower root/shoot ratio than non-leguminous forbs and legumes (root/shoot ratios based on dry matter across all harvests were 0·44, 0·65 and 0·52 for grasses, forbs and legumes, respectively). This would mean a greater nutrient allocation to below-ground plant parts and therefore greater ^15^N and ^13^C allocation to roots. Moreover, grass root systems usually also have less ligneous structures and higher turnover rates than other root systems, leading to a more rapid incorporation of C and N into root systems (Gross, Maruca, & Pregitzer 1992; Eissenstat 2000). This is also supported by the study of Hertenberger & Wanek (2004) showing that the ^15^N signal after commencement of labelling with stem infiltration reached the roots of the grass *L. perenne* after 20 hours, but only after 26 hours in the forb *Centaurea jacea*. Despite higher root biomass than shoot biomass, the forb *P. lanceolata* and the legume *L. corniculatus* allocated only very small amounts of ^15^N and ^13^C into their roots, indicating very little turnover of the rather course root systems of these species.

Considering the more general patterns of ^15^N enrichment, species allocated on average 74%^15^N into shoots, which indicates that a substantial amount was transported into their root systems. The 26% isotope allocation into roots is in contrast to other studies mainly of crop plants suggesting that only a small fraction of the label taken up foliarly was transferred into roots (Palta *et al.* 1991; Russell & Fillery 1996; McNeill, Zhu, & Fillery 1997; Schmidt & Scrimgeour 2001; Khan, Peoples, & Herridge 2002). These contrasting findings perhaps reflect morphological and physiological differences between grassland plants and crops manifested by different growth patterns and a higher allocation of resources into the root system by grassland plants. Species of less productive grasslands are expected to have lower growth rates as well as greater partitioning of photosynthates and nutrients to root systems for effective water and nutrient uptake under conditions of competition and stress (Chapin 1980). In contrast, crop plants are bred for fast growth, which is mainly the result from biomass allocation to leaf biomass but not into root systems.

Our finding of a considerably lower ^13^C enrichment than ^15^N enrichment in shoots and roots across all species is consistent with other studies (Schmidt & Scrimgeour 2001), reflecting the facts that urea [CO(NH_2_)_2_] provides two atoms of N for each atom of C and that some ^13^C is lost through respiration (Lakkineni *et al.* 1995). Nevertheless, it was still interesting to see that ^13^C in urea solution applied onto the leaf surface can enter the plant tissue and is incorporated into the root system. Schmidt & Scrimgeour (2001) discuss some evidence suggesting that urea-derived C and atmospheric C, once taken up by leaves, are assimilated and translocated in a similar fashion. Another possible reason for restricted ^13^C enrichment is the over-supply of N to the plant, which may lead to reduced carbohydrate accumulation (Gooding & Davies 1992); however, this is unlikely in our system as the soil nutrient concentrations were only moderate. Among legumes, only 7%^15^N of the whole-plant isotopic signal were allocated into roots, suggesting that rhizobia associated with all tested legume species might have diluted the ^15^N signal in roots. The ^13^C enrichment of legume roots was perhaps lower than that of other plants because of the contribution of C stored in existing root nodule biomass before labelling commenced (nodule biomass was not measured separately in this study). An exception to the ^13^C enrichment patterns was the legume *T. pratense* that showed a four-times higher ^13^C enrichment in shoot than all other species. We explain this pattern by the small growth and biomass increase of this species, leading to an accumulation of the isotopic label in the shoots with only little transfer to the roots. Moreover, in stable isotope studies, there is always the possibility of sample contamination, but it is unlikely that all three sample replicates were contaminated.

### Persistance of the labelling signal

No data on the persistence of the labelling signal after foliar feeding are available for grassland plant species. With the proposed method, isotopic enrichment levels in shoots and roots were generally low for both ^15^N and ^13^C (<1% APE) and these levels either decreased (e.g. grass shoots), increased (e.g. legume shoots) or remained unchanged (e.g. forb shoots) over 4 weeks with re-labelling each week. Enrichment levels observed here are similar to those achieved in the native forb *C. jacea* or the grass *Lolium perenne* using the leaf tipping method measured 48 hours after labelling (Hertenberger & Wanek 2004). To increase the isotopic signal in the tested plant species, a higher concentration of the urea solution would be necessary; however, this could increase the risk of causing leaf burning damage (Bremner 1995). This need to use low urea concentrations also limits the maximum ^13^C enrichment achievable with urea leaf-feeding. More than one labelling a week would probably sustain a higher isotopic signal over a longer period as indicated by the negative correlation between isotopic signal and shoot mass or leaf area, however, whether this varies among species requires further testing.

In conclusion, the simple dual-labelling method tested in the current study appears to be a feasible alternative to growing plants in enriched soil and gas-tight laboratory chambers or using portable labelling enclosures. Together with other recent developments in isotope labelling (e.g. plant seed labelling, Carlo, Tewksbury, & Martinez del Rio 2009), the current method opens new avenues for studying ecological interactions *in situ* in plant communities. In particular, the method can be used to identify linkages between specific plants in a community and soil organisms and to quantify the contributions of individual plant species to soil processes linked to C and N inputs (e.g. Orwin *et al.* 2010; Witt & Setälä 2010).

## Supporting Information

**Table S1.** Dry mass, N and C concentration of shoots and roots of 12 native grassland species comprising the functional groups grasses, non-leguminous forbs and leguminous forbs after one week and after four weeks of foliar labelling (label.).

As a service to our authors and readers, this journal provides supporting information supplied by the authors. Such materials may be re-organized for online delivery, but are not copy-edited or typeset. Technical support issues arising from supporting information (other than missing files) should be addressed to the authors.
