# Supplementary material for: A simple method for in situ-labelling with 15N and 13C of grassland plant species by foliar brushing
Source: Methods Ecol Evol. 2011 Jun;2(3):326–32. doi: 10.1111/j.2041-210X.2010.00072.x (PMC3573864; doi:10.1111/j.2041-210X.2010.00072.x)
Supplement: Supplementary file 1 [file mee30002-0326-SD1.doc]

Table S1. Dry mass, N and C concentration of shoots and roots of 12 native grassland species comprising the functional groups grasses, non-leguminous forbs and leguminous forbs after one week and after four weeks of foliar labelling (label.). Missing values due to failure of germination. Means, n=3.

Week one Week four

Funct. Plant Plant Dry mass (mg) N conc. (%) C conc. (%) Dry mass (mg) N conc. (%) C conc. (%)

group species part

Control Label. Control Label. Control Label. Control Label. Control Label. Control Label.

Grasses *A. elatius* Shoot 74.2 84.9 5.2 4.7 42.8 42.1 154.2 206.1 4.3 4.3 45.8 42.4

Root 28.6 21.7 2.7 1.8 38.9 37.7 29.5 115.4 1.6 1.2 46.2 43.2

*B. media* Shoot 37.2 78.3 3.0 2.6 49.1 29.7 Root 2.3 13.5 1.2 1.7 49.2 42.2 *B. erectus* Shoot 80.1 36.2 4.7 4.2 41.5 40.3 240.7 259.3 3.3 3.7 47.0 41.6

Root 37.5 16.8 1.5 2.1 31.5 36.4 159.9 161.4 1.5 1.7 42.5 37.9

*D. glomerata* Shoot 84.6 74.2 4.4 4.5 40.6 40.8 220.2 214.8 4.0 4.2 46.8 41.6

Root 31.0 28.5 1.4 1.8 42.4 39.6 154.2 80.7 1.4 2.1 47.8 41.9

Forbs *K. arvensis* Shoot 23.0 21.3 3.3 4.2 43.0 45.4 26.1 32.8 2.8 4.4 38.9 39.6

Root 11.1 19.1 2.7 3.9 46.2 42.9 18.5 17.7 1.6 2.9 48.7 46.6

*L. ircutianum* Shoot 30.6 69.8 3.4 3.5 45.7 39.9 107.6 3.5 40.2

Root 7.3 19.7 2.8 2.2 46.6 41.3 56.0 3.0 40.2

*P. lanceolata* Shoot 18.3 22.9 4.5 3.9 41.4 42.2 84.4 97.4 3.4 3.7 46.2 41.5

Root 22.2 28.7 3.0 5.1 36.1 40.4 55.7 74.5 1.8 2.5 46.4 41.3

*R. obtusifolius* Shoot 295.5 498.8 3.8 4.6 44.3 40.2 356.3 619.8 3.5 3.4 42.8 38.8

Root 350.8 288.1 2.1 2.4 46.0 41.3 115.2 589.9 1.7 2.2 45.3 40.0

*S. pratense* Shoot 22.7 21.2 5.0 4.5 41.5 41.1 20.3 37.0 3.5 3.6 40.8 37.0

Root 15.6 17.0 3.2 3.9 42.3 40.7 17.9 25.9 3.1 3.0 45.5 45.2

Legumes *L. corniculatus* Shoot 5.8 8.1 5.0 7.6 47.1 42.1 8.3 5.6 5.8 7.1 43.7 46.1

Root 8.6 9.9 3.8 6.5 44.3 38.6 6.4 4.5 1.6 3.6 40.4 45.9

*M. lupulina* Shoot 13.8 11.3 4.1 3.1 46.3 39.6 91.1 67.6 3.1 3.6 46.4 42.1

Root 4.7 5.8 2.1 3.2 49.9 41.7 60.2 33.0 3.0 4.1 46.5 41.1

*T. pratense* Shoot 8.3 7.6 6.1 7.3 46.1 42.8 13.5 19.6 5.7 3.6 48.6 33.5

Root 6.5 7.2 2.8 3.4 45.1 40.0 6.8 8.7 2.6 2.6 50.0 44.5
